# KBES: A dataset for realistic Bangla speech emotion recognition with intensity level

**DOI:** 10.1016/j.dib.2023.109741

**Published:** 2023-10-31

**Authors:** Md. Masum Billah, Md. Likhon Sarker, M. A. H. Akhand

**Affiliations:** Department of Computer Science and Engineering, Khulna University of Engineering & Technology (KUET), Bangladesh

**Keywords:** Bangla speech, Speech emotion recognition, Intensity level

## Abstract

Speech Emotion Recognition (SER) identifies and categorizes emotional states by analyzing speech signals. SER is an emerging research area using machine learning and deep learning techniques due to its socio-cultural and business importance. An appropriate dataset is an important resource for SER related studies in a particular language. There is an apparent lack of SER datasets in Bangla language although it is one of the most spoken languages in the world. There are a few Bangla SER datasets but those consist of only a few dialogs with a minimal number of actors making them unsuitable for real-world applications. Moreover, the existing datasets do not consider the intensity level of emotions. The intensity of a specific emotional expression, such as anger or sadness, plays a crucial role in social behavior. Therefore, a realistic Bangla speech dataset is developed in this study which is called KUET Bangla Emotional Speech (KBES) dataset. The dataset consists of 900 audio signals (i.e., speech dialogs) from 35 actors (20 females and 15 males) with diverse age ranges. Source of the speech dialogs are Bangla Telefilm, Drama, TV Series, Web Series. There are five emotional categories: Neutral, Happy, Sad, Angry, and Disgust. Except Neutral, samples of a particular emotion are divided into two intensity levels: Low and High. The significant issue of the dataset is that the speech dialogs are almost unique with relatively large number of actors; whereas, existing datasets (such as SUBESCO and BanglaSER) contain samples with repeatedly spoken of a few pre-defined dialogs by a few actors/research volunteers in the laboratory environment. Finally, the KBES dataset is exposed as a nine-class problem to classify emotions into nine categories: Neutral, Happy (Low), Happy (High), Sad (Low), Sad (High), Angry (Low), Angry (High), Disgust (Low) and Disgust (High). However, the dataset is kept symmetrical containing 100 samples for each of the nine classes; 100 samples are also gender balanced with 50 samples for male/female actors. The developed dataset seems a realistic dataset while compared with the existing SER datasets.

Specifications Table

**Table:** 

Subject	Signal processing
Specific subject area	Speech emotion recognition, emotion classification with intensity level
Type of data	Audio files
How the data were acquired	Videos were collected from social media platforms (Facebook and YouTube). Emotion speech dialogs were extracted from the videos using VideoProc Converter software. Each video clip was converted to standard audio format using Any Video Converter software where the duration of the video clip is 3 s. The tools used are: • Dell Inspiron Laptop • HP Pavilion Laptop • Headset • VideoProc Converter^1^ • Any Video Converter^2^
Data format	Raw and analyzed Waveform Audio File Format (WAV)
Description of data collection	Raw video samples were splitted according to five different emotional states: Neutral, Happy, Sad, Angry, and Disgust. Each emotional sample (except Neutral) was classified either Low or High based on the intensity of the emotion. Total 900 emotional speeches were selected for the KBES dataset. Dataset is balanced symmetrically containing equal number of audio samples for each emotion category. Samples are also gender balanced in each emotion category having equal number of samples for male and female.
Data source location	Bangla social media flatforms, Bangladesh Primary sources: https://www.facebook.com/ and https://www.youtube.com/
Data accessibility	Repository name: Mendeley Data Digital object identifier: 10.17632/vsn37ps3rx.4 URL to data: https://data.mendeley.com/datasets/vsn37ps3rx
Related research article	The KBES dataset is a part of the research “Bangla Speech Emotion Recognition with Intensity Level using Feature Transformation and Deep Learning”. An article with the same title is under preparation and will be submitted soon in a suitable prestigious journal.

1 www.videoproc.com.

2 www.any-video-converter.com.

## 1 Value of the Data

• The developed KUET Bangla Emotional Speech (KBES) dataset contains a unique collection of Bangla audio speech for realistic Bangla Speech Emotion Recognition (SER). Bangla has more than 300 million speakers world-wide. However, Bangla audio speech datasets for emotion recognition are very limited; and, all of those are recorded in studio for predefined dialogs by selected actors. Such datasets for predefined limited dialogs might not be effective for real-life SER applications. On the other hand, the developed KBES dataset is the collection of cropped emotional contents from Bangla Telefilm, Drama, TV Series and Web Series. As the individual dialogs are diverged (not defined, nor repetitive), the KBES is a realistic dataset compared to the existing datasets.
• The KBES dataset covers audio speech of foremost emotion states Neutral, Happy, Sad, Angry and Disgust. Therefore, the dataset is useful for real-life applications as a five-class classification problem. The actors of the KBES dataset are diverged especially in the age context; there are total of 35 actors whose age varied between 15 and 70 years. However, the dataset is balanced with an equal number of male and female speeches for each emotion.
• The developed KBES dataset is unique to the level of emotion intensity along with classification which has remarkable prospects [1]. It is notable that intensity of a particular emotion has an impact on real-life activity. More specifically, a high-level emotional intensity of Sad, Angry or Disgust may lead a person to engage in destructive activity (e.g., suicidal event). Individual speech of Happy, Sad, Angry and Disgust are marked as Low or High in the KBES dataset. Therefore, the KBES is useful for developing emotion intensity based practical Bangla SER system where the existing datasets are not suitable.

## 2 Objective

SER is an emerging research area due to its importance in social, cultural, and business domain. A suitable dataset is an important resource for SER related studies in a particular language. There is an apparent lack of SER datasets in the Bangla language although it is one of the most spoken languages in the world. There are a few Bangla SER datasets but those consist of only a few dialogs with a minimal number of actors making them unsuitable for real-world applications. Moreover, the existing datasets do not consider the intensity level of emotions. The intensity of a specific emotional expression, such as anger or sadness, plays a crucial role in social behavior. Therefore, a realistic Bangla speech dataset is developed in this study which is called KUET Bangla Emotional Speech (KBES) dataset. The KBES dataset will contribute to enhance Bangla SER and human-computer interaction research.

## 3 Data Description

SER identifies and categorizes emotional states by analyzing speech signals. SER is a language specific research; and an appropriate dataset is an important issue for SER related studies in a particular language. The developed KBES dataset is a realistic Bangla emotional speech dataset and Table 1 provides a descriptive summary of the dataset.

**Table 1:** A summary of the KBES dataset.

Descriptions	Values
Speech Source	Bangla Telefilm, Drama, TV Series, Web Series
Used language	Standard Bangla
Dataset type	Realistic
File type	Audio only
File format of audio clips	WAV
Sampling rate (video clip)	44.1 kHz
Sampling rate (audio clip)	48 kHz
Emotion states	Neutral, Happy, Sad, Angry, Disgust
Intensity levels	Low, High
Number of actors	35
Total number of audio clips	900
Number of speech dialogs	Almost 900
Audio duration per clip	3 S
Total duration of the dataset	2700 S
Size of the dataset	497 MB
Utilized software	VideoProc Converter, Any Video Converter

KBES dataset contains samples of five emotional categories: Neutral, Happy, Sad, Angry, and Disgust. Except Neural, each category has two intensity levels (Low and High). Two different folders contain Low and High intensity samples for each of Happy, Sad, Angry, and Disgust emotions. For examples, Happy (Low) and Happy (High) folders contain Happy emotion samples in Low and High intensity categories, respectively. Each of the folder contains 100 samples of the corresponding intensities. There are 200 audio samples for each of Happy, Sad, Angry and Disgust. On the other hand, Neutral emotion has no intensity level and the Neutral folder contains 100 audio samples. There are total of 900 (200 **×** 4 + 100) audio samples in nine folders in KBES dataset. The audio clips are in standard WAV format where frequency of each of the audio is 48 kHz.

**Fig. 1:**
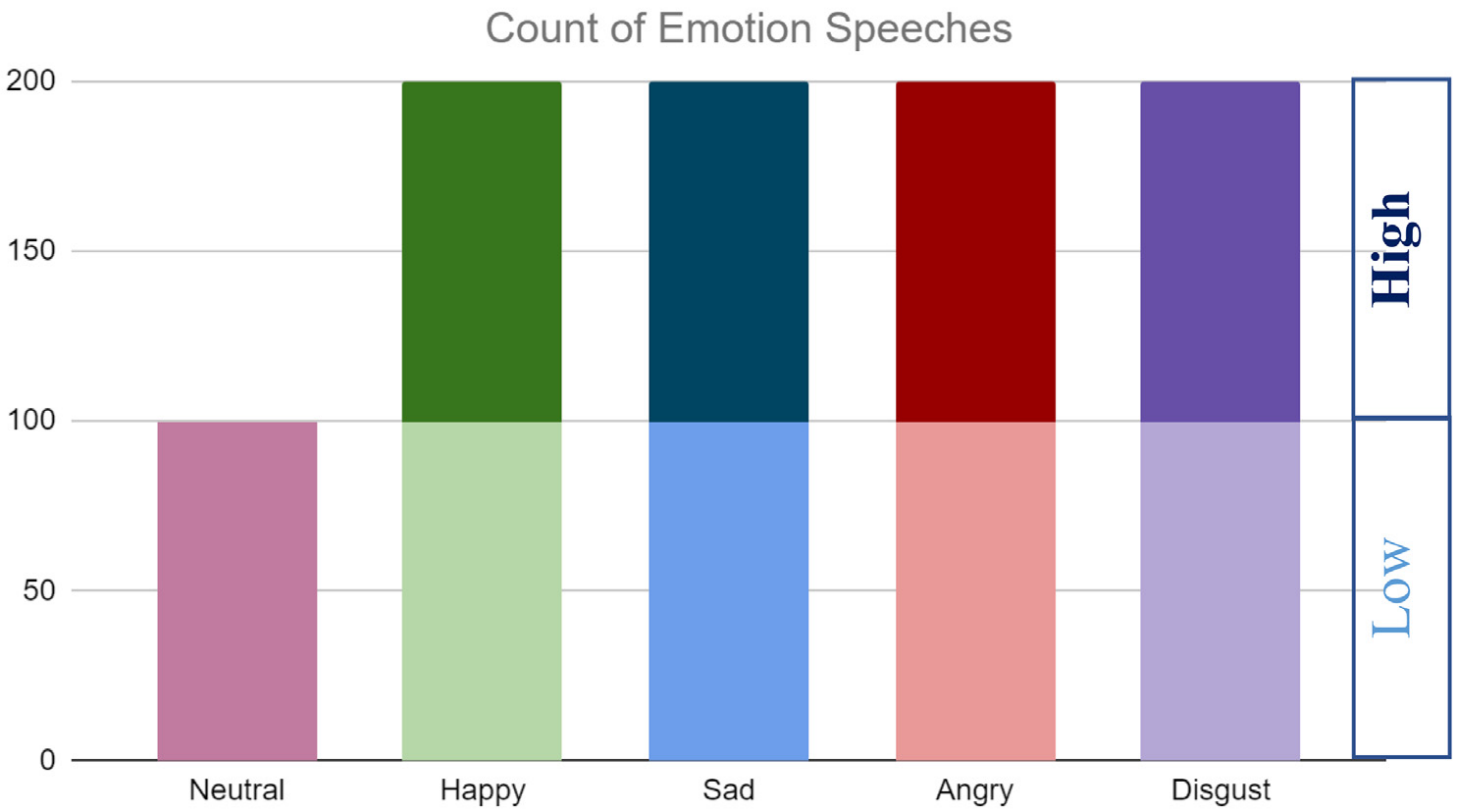
Count of emotion samples in the KBES dataset.

The KBES dataset is balanced in terms of male-female speeches and per emotion category samples. The dataset is gender-balanced having equal total 450 samples for male and female actors. For a particular emotional category, speeches are also balanced having 50 samples from male and female actors. As an example, Happy (Low) folder contains 100 samples where 50 samples are collected from female speeches and the remaining 50 samples are male speeches. Fig. 1 shows emotion category-wise sample counts in the developed KBES dataset.

The developed KBES dataset, having intensity measures for different emotional states, might be valuable for better Bangla SER research. The intensity level of each speech of Happy, Sad, Angry, or Disgust is identified as either Low or High. To make a realistic dataset with diverse speech samples, speeches from 35 different actors were selected after rigorous analysis of a large number of video samples. Table 2 illustrates the actor wise emotion sample counts; the first 20 rows in the table are for female actresses, and the remaining 15 rows are for male actors. It is notable from the table that the number of samples are not same for individual actors. Moreover, individual actors do not have samples for all the emotion categories. The reason behind that the suitable samples for all the emotional categories are not found in the collected video samples. As an example, there are 21 samples in four different emotion categories for the actress Afsana Mimi; those are Neutral, Happy (Low), Sad (Low), and Disgust (High) having samples eight (8), seven (7), five (5), and one (1), respectively. The highest number of samples for actress Tanjin Tisha is 155 for seven (7) emotion categories; no samples are available for the Neutral and Angry (Low) categories. It is notable that individual dialogs in the KBES dataset are distinct for a particular emotion category and even for an individual actor. More significantly, samples of the dataset are not collected in laboratory environment for SER purposes like other available datasets. At a glance, all 900 samples are almost distinct. Such a dataset with diversity in speeches can be used in situations like human-machine interaction, research domain, and identity verification.

**Table 2:** Actor wise emotion sample counts in the KBES dataset; the first 20 rows are for female actresses and the remaining 15 rows for the male actors.

SL	Name	Neutral	Happy (Low)	Happy (High)	Sad (Low)	Sad (High)	Angry (Low)	Angry (High)	Disgust (Low)	Disgust (High)	Total
1	Afsana Mimi	8	7		5					1	**21**
2	Dilara Zaman	8									**8**
3	Dolon Dey		2			12					**14**
4	Jatri		2								**2**
5	Keya Akter Payel		10	23						23	**56**
6	Lucky Enam	13			9		5			5	**32**
7	Lutfun Nahar						6				**6**
8	Masuda Sharfuddin						4				**4**
9	Mehazabien Chowdhury							9			**9**
10	Purnima			3			17				**20**
11	Quazi Nawshaba Ahmed			11							**11**
12	Sabila Nur			2							**2**
13	Safa Kabir			8			8				**16**
14	Sara Zaker	4	2								**6**
15	Shaila Sabi								12		**12**
16	Shila Ahmed		4						3		**7**
17	Shomi Kaiser	14	4		7		10			1	**36**
18	Suborna Mustafa	3	1		9				4	8	**25**
19	Tanjin Tisha		17	3	20	38		34	31	12	**155**
20	Tasnia Farin		1					7			**8**
21	Abul Hayat	15	6		2		32	35			**90**
22	Abdul Kader	3	6	2	3		18	15			**47**
23	Afran Nisho		14	30					48	20	**112**
24	Asaduzzaman Noor	5	12		4						**21**
25	Azizul Hakim	10	1		8						**19**
26	Farhan Ahmed Jovan			8	3	17				15	**43**
27	Humayun Faridi									6	**6**
28	Manoj Kumar Pramanik			4		17			2		**23**
29	Mozammel Hossain			2	4					6	**12**
30	Rahmat Ali		2							3	**5**
31	Sharaf Ahmed Jibon		4								**4**
32	Tanzim Hasan Anik		3	3							**6**
33	Tawsif Mahbub		2								**2**
34	Zahid Hasan	17		1							**18**
35	Ziaul Faruq Apurba				26	16					**42**

	**Total:**	**100**	**100**	**100**	**100**	**100**	**100**	**100**	**100**	**100**	**900**

The developed KBES dataset is significant compared to other existing SER datasets, especially for Bangla. A comparison of the existing prominent SER datasets with the KBES dataset is shown in Table 3. Most of the datasets are laboratory curated; those are developed with a few fixed numbers of speech dialogs those repeatedly read out acting different emotions in a laboratory environment by a small number of actors or research volunteers. Again, most of existing datasets are in English. Some well-known publicly available laboratory curated benchmark datasets are IEMOCAP [2], RAVDESS [3], SAVEE [4], and EmoDB [5]. Except laboratory curated ones, material sources of EmoFilm [6], VESUS [7] and EmoSpeech [8] are film or in wild. Only a few laboratory curated datasets are available for Bangla language, such as SUBESCO [9] and BanglaSER [10]. As an example, the 7000 samples of popular Bangla SUBESCO [9] dataset are developed with only 10 speech dialogs repeatedly reading by 20 actors. Besides, only three dialogs were used to prepare 1467 samples in BanglaSER [10] dataset. Therefore, the existing datasets do not contain sample which reflect real-life scenario. In contrast, the KBES dataset is developed extracting emotional dialogs from various sources (like Drama, TV series), not acting on pre-defined dialogs. Thus, the developed KBES dataset contains 900 samples with almost unique dialogs. Such dataset provides a diverse representation of emotions and helps to recognize emotion from a realistic environment. Although the developed KBES contains relatively small number of samples with respect to several existing datasets (especially laboratory curated and wild type cases), KBES contains significant diversity in samples as it is developed from relatively large number actors and dialogs.

**Table 3:** A comparative summary among different public SER datasets with the developed KBES dataset.

Dataset	Number of Samples	Number of Dialogs	Number of Emotions	Intensity with Emotion	Number of Actors	Sampling Rate	Class Balance	Gender Balance	Language	Material Source
IEMOCAP [2]	5255	3	9	No	10	16 kHz	Yes	Yes	English	Lab curated
RAVEDESS [3]	1440	2	8	Yes	24	48 kHz	No	Yes		Lab curated
SAVEE [4]	480	15	7	No	4	44.1 kHz	No	No		Lab curated
EmoDB [5]	535	10	7	No	10	16 kHz	No	Yes	German	Lab curated
EmoFilm [6]	1115	111	5	No	NA	48 kHz	No	No	English, Italian, Spanish	Film
VESUS [7]	12,594	252	5	No	10	NA	Yes	Yes	English	In wild
EmoSpeech [8]	8000	6	4	No	250	16 kHz	No	No	English and Hindi	In wild
SUBESCO [9]	7000	10	7	No	20	48 kHz	Yes	Yes	Bangla	Lab curated
BanglaSER [10]	1467	3	5	No	34	44.1 kHz	Yes	Yes		Lab curated
Developed KBES	900	900	9	Yes	35	48 kHz	Yes	Yes		YouTube, Facebook

Most significantly, KBES dataset contains emotion category with the intensity level (Low or High) which is the unique in Bangla SER domain. Since intensity level for a particular emotion (e.g., Disgust) has great influence on human behavior [1], the dataset has exposed to a new research direction in Bangla. On the other hand, the developed KBES dataset is balanced in samples per emotion category and male-female speech which is important to use the dataset in machine learning applications. The sample wise balanced dataset preparation with the diverse, realistic, and unique dialogs was a challenging issue, which is a major contribution in the KBES dataset development.

## 4 Experimental Design, Materials and Methods

**Fig. 2:**
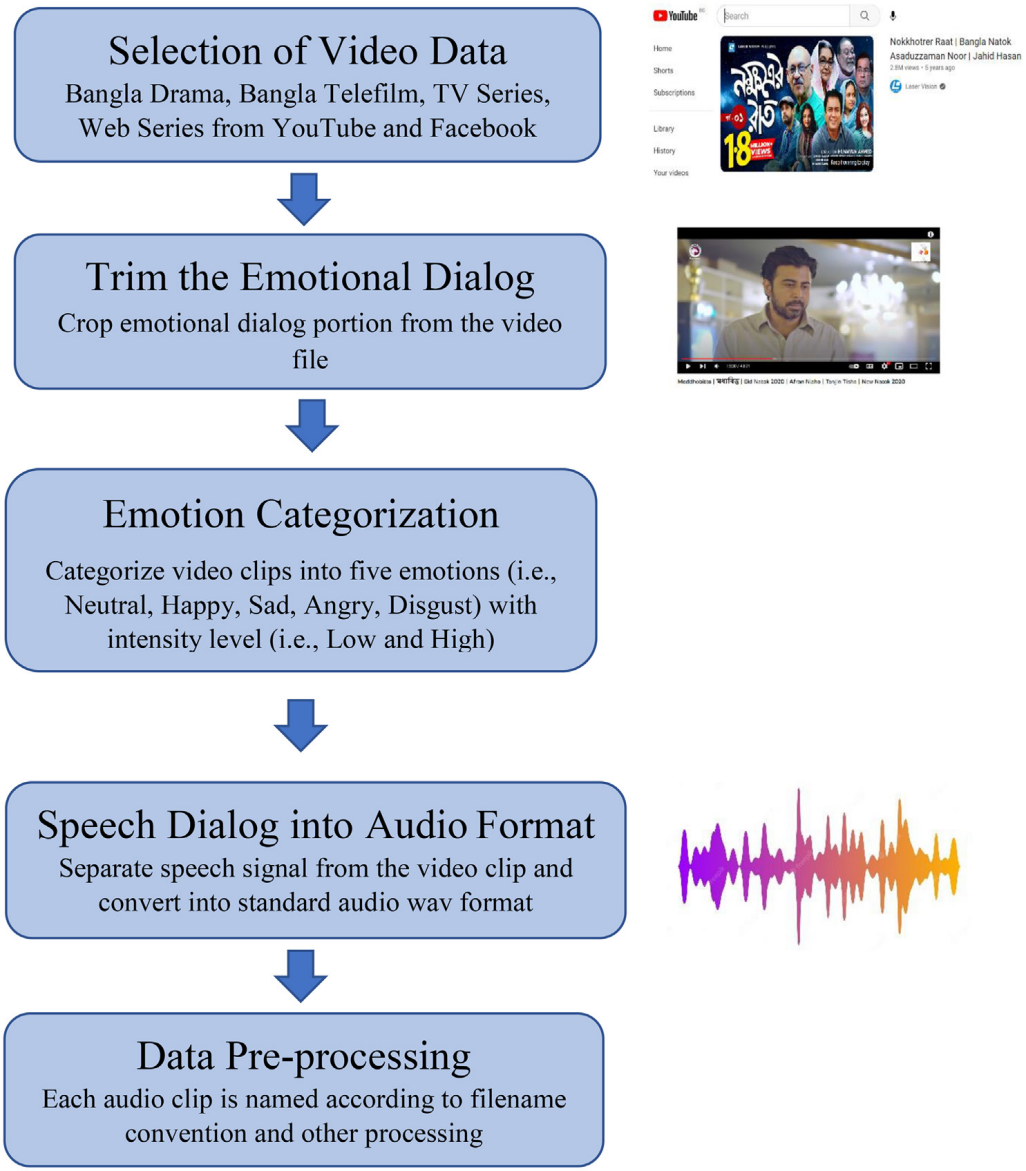
Workflow diagram of the KBES dataset preparation.

The process of KBES dataset preparation consists of five major steps illustrates in Fig. 2. The following subsections briefly describe the major steps.

### 4.1 Collection of raw videos

Initially, publicly available Bangla Drama, Telefilm, TV Series, Web Series videos were collected from YouTube and Facebook platforms. YouTube is also the main source of Facebook videos through a given link. Most of the cases, video files were collected in series, i.e., collected all the series of a drama. More than 5000 video clips were collected and analyzed for preparation of the dataset.

### 4.2 Selection of emotions and trimming videos into emotional dialogs

All the process of collecting videos from raw sample to the final emotion samples are done manually by listening the clips multiple times. It is identified that Happy, Sad, Angry, and Disgust are more exposable through speech than other emotions like Anxiety. Therefore, including Neutral, five emotional categories (i.e., Neutral, Happy, Sad, Angry or Disgust) have been considered for the KBES dataset. Every individual video clip is trimmed to the emotional dialog portion from the videos. More than 3000 emotional dialogs were trimmed for the next step emotion categorization.

### 4.3 Emotion categorization considering intensity level

All the video clips were analyzed manually and considered five emotion related clips for processing. Every emotion speech is also classified again according to intensity level (i.e., Low or High). An emotion clip with high frequency is considered in high intensity level and it was decided by listening the video clip manually. For example, an Angry emotional clip was classified as either high or low intensity level based on its frequency and rudeness. Three native Bangla speakers (i.e., authors) were directly involved throughout the dataset preparation. One author first listened video clip and categorized as emotion (i.e., Neutral, Happy, Sad, Angry or Disgust) plus intensity level (i.e., Low or High). Another author individually listened and verified the labeling. Third author also reviewed whole process closely, and randomly checked several samples. Authors ensures the quality and correctness of the video clips for each intensity level. Among the collected emotional video clips, selected 900 videos clips were found most suitable and fits for the intensity level. Thus, KBES holds 900 emotional audio speeches separating from the categorized video clips.

### 4.4 Conversion from video to audio

The source bitrates of the videos downloaded from YouTube, Facebook is 44.1 kHz. VideoProc Converter is utilized to trim each video recording clip to 3 s. These video clips are then converted to WAV audio format using Any Video Converter software where frequency of the audio signal is 48 kHz. Therefore, the duration of each speech is precisely 3 s, and the converted voice doesn't distort much from the actual voice. As a result, the size of the total audio files decreases, and it is 497 MB after converting video speeches to audio format.

### 4.5 Data pre-processing

Finally, pre-processing is performed on the audio clips to fit those for machine learning applications. At this stage, each audio clip is named according to filename convention. Each data file is assigned to a unique filename with fully anonymized the actors. Description of the renaming process is shown in Table 4. Five different emotion categories have been defined with numeral from 1 through to 5 for Neutral, Happy, Sad, Angry and Disgust emotions. Similarly, intensity is represented using two numbers: 1 for Low intensity level and 2 for High intensity level. To include gender identity with sample, female is tagged by number 0 and male is represented by number 1. Individual speeches are also uniquely marked with a three (03) digits number and starting from 001. The identifiers are ordered as “Emotion Category - Intensity Level - Gender - Speech number.wav”. For example, the filename “2–1–0–001.wav” refers to “Happy-Low-Female-1st speech”, and the filename “3–2–1–002.wav” refers to “Sad-High-Male-2nd Speech”.

**Table 4:** Description of the filename convention.

Identifier	Meaning
State of Emotion	1 = Neutral, 2 = Happy, 3 = Sad, 4 = Angry, 5 = Disgust
Intensity Level of Emotion	1 = Low, 2 = High
Gender	0 = Female, 1 = Male
Speech Number	001 = 1st Speech, …, 050 = 50th Speech

## Ethics Statements

Emotional speech dialogs are extracted from publicly available Telefilm, Drama, TV Series and Web Series in YouTube and Facebook. YouTube is also the main source of Facebook videos through the given link. YouTube videos are usable for derivative works through the license to other users. Authors ensure that there are no copyright issues on video clips used in this study to prepare the speech emotion dataset. Individually, all the actors are professional and dialogs were performed for public audience acting in Telefilm, Drama, TV Series and Web Series. Moreover, actors have been fully anonymized in the speech dialog data samples of the developed dataset.

## CRediT authorship contribution statement

**Md. Masum Billah:** Conceptualization, Methodology, Software, Funding acquisition, Data curation, Validation, Writing – original draft. **Md. Likhon Sarker:** Conceptualization, Methodology, Software, Funding acquisition, Writing – original draft. **M. A. H. Akhand:** Supervision, Writing – review & editing, Investigation.

## Data Availability

KUET Bangla Emotional Speech (KBES) Dataset (Original data) (Mendeley Data)
